# Supplementary material for: Proton pumping accompanies calcification in foraminifera
Source: Nat Commun. 2017 Jan 27;8:14145. doi: 10.1038/ncomms14145 (PMC5290161; doi:10.1038/ncomms14145)
Supplement: Supplementary Information — Supplementary figures and supplementary references. [file ncomms14145-s1.pdf]

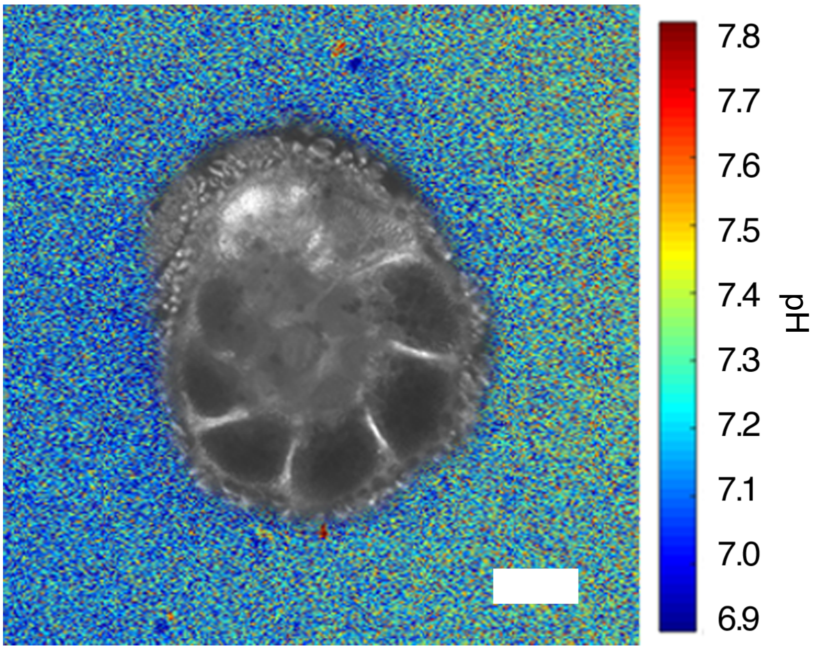

Supplementary Figure 1: The effect of a V-type H^+ ATPase inhibitor on external pH. Two-dimensional variability in pH around the specimen during production of a new chamber in the presence of 1µM Bafilomycin A1 (specimen No. 13 in Table 1). No distinguishable pH gradation can be observed though the picture was taken though the timing of the lowest pH (8h05m.). Scale bar indicates 50 µm.

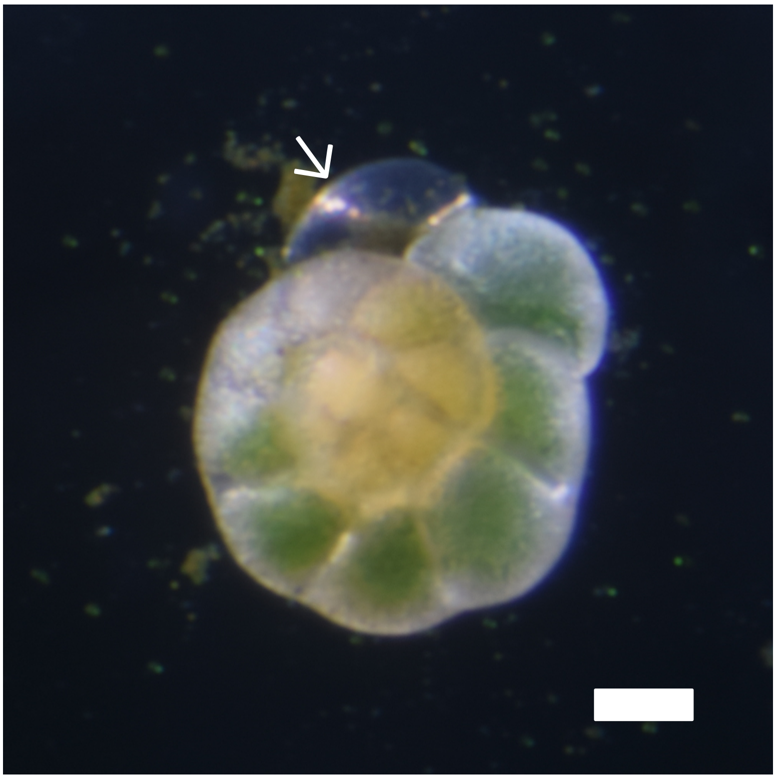

Supplementary Figure 2: The effect of a V-type H^+ ATPase inhibitor on chamber formation. Newly added chamber under 1µM Bafilomycin seawater (specimen No. 13 in Table 1). Only a thin wall (indicated by white arrow) was built over a time period of 8 hours. Scale bar indicates 50 µm.

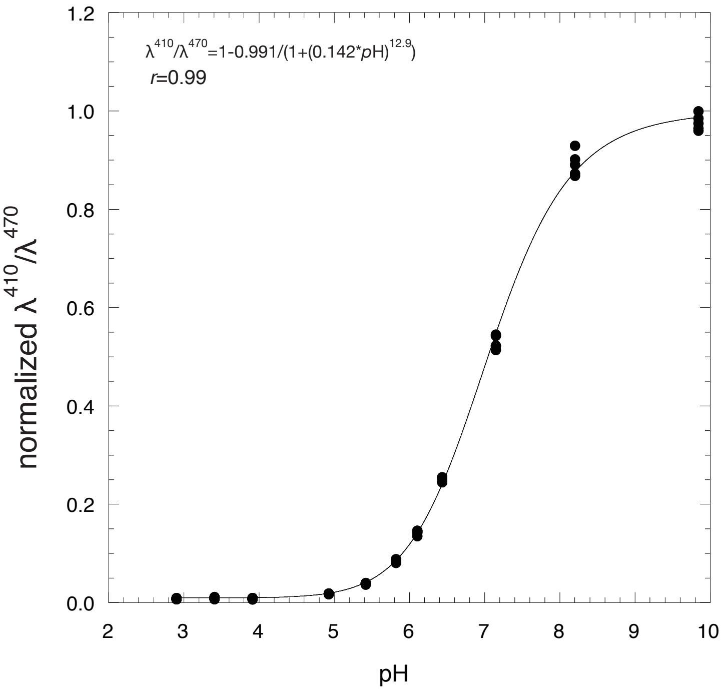

Supplementary Figure 3: Optimized calibration curve of HPTS with various pH conditions. Optimized calibration curve of HPTS with various pH conditions. For most data points, standard deviations are smaller than the size of the symbols. The pH-normalized relation was found to follow the Boltzmann function. To relate the λ_2^{exc}/ λ_1^{exc} ratio to seawater pH, a 11-step calibration set was prepared by adjusting the pH of 20 µM HPTS in natural seawater with 0.1 M sodium hydroxide (NaOH) or 0.1 M hydrochloric acid (HCl). The same set was also captured with the two neutral density filters occasionally used to reduce the emission intensity. This curve is similar to that previously published for similar conditions and application[1].

Supplementary References

1 De Nooijer, L. J., Toyofuku, T., Oguri, K., Nomaki, H. & Kitazato, H. Intracellular pH distribution in foraminifera determined by the fluorescent probe HPTS. *Limnology and Oceanography: Methods* 6, 610-618, doi:10.4319/lom.2008.6.610 (2008).
